# A Bacterial Dynamin-Like Protein Confers a Novel Phage Resistance Strategy on the Population Level in Bacillus subtilis

**DOI:** 10.1128/mbio.03753-21

**Published:** 2022-02-15

**Authors:** Lijun Guo, Laura Sattler, Samia Shafqat, Peter L. Graumann, Marc Bramkamp

**Affiliations:** a Ludwig-Maximilians-Universität München, Faculty of Biology, Planegg-Martinsried, Germany; b SYNMIKRO, LOEWE-Zentrum für Synthetische Mikrobiologie, Marburg, Germany; c Fachbereich Chemie, Universität Marburg, Marburg, Germany; d Institute for General Microbiology, Christian-Albrechts-University Kiel, Kiel, Germany; e Central Microscopy Facility, Christian-Albrechts-University Kiel, Kiel, Germany; Nanyang Technological University

**Keywords:** phage, bacterial dynamin-like protein, DynA, ɸ29, SPβ, membrane integrity, lysis

## Abstract

Bacillus subtilis DynA is a member of the dynamin superfamily, involved in membrane remodeling processes. DynA was shown to catalyze full membrane fusion and it plays a role in membrane surveillance against antibiotics. We show here that DynA also provides a novel resistance mechanism against phage infection. Cells lacking DynA are efficiently lysed after phage infection and virus replication. DynA does not prevent phage infection and replication in individual cells, but significantly delays host cell lysis, thereby slowing down the release of phage progeny from the host cells. During the process, DynA forms large, almost immobile clusters on the cell membrane that seem to support membrane integrity. Single-molecule tracking revealed a shift of freely diffusive molecules within the cytosol toward extended, confined motion at the cell membrane following phage induction. Thus, the bacterial dynamins are the first anti-phage system reported to delay host cell lysis and the last line of defense of a multilayered antiviral defense. DynA is therefore providing protective effects on the population, but not on single cell level.

## INTRODUCTION

The dynamin-like protein (DLP) DynA of Bacillus subtilis is a member of the dynamin superfamily of proteins. DynA forms an unusual two-headed DLP that arose through a gene fusion in firmicute bacteria (1). Consequently, DynA contains two GTPase domains. The protein is involved in membrane remodeling processes and was shown to tether membranes in *trans* to promote full membrane fusion (1, 2). *In vitro* experiments have shown that DynA binds GTP to both nucleotide-binding domains and both domains need to be loaded with GTP to activate hydrolysis (1). GTP-binding or hydrolysis is not required for membrane tethering and lipid mixing, only full membrane fusion (content mixing) was slightly accelerated when GTP was present (2). Thus, the precise role of the nucleotide hydrolysis activity of DynA remained somewhat obscure. Deletion of the *dynA* allele in B. subtilis leads to a stress-sensitivity phenotype. We have recently shown that DynA plays a role in membrane surveillance (3). The protein is highly mobile at the membrane, but clusters into large and confined assemblies upon addition of the pore-formation antibiotic nisin. Therefore, we hypothesized that DynA is recruited to sites of damaged membranes (3). Furthermore, a genetic link between DynA and the membrane organizing flotillin protein has been shown (4), supporting the notion that DynA is involved in membrane remodeling processes. We have previously reported that the B. subtilis phage ɸ29 can form more plagues on the lawn of DynA-deficient strain compared with wild-type strain (3). ɸ29 is the smallest phage known to infect B. subtilis so far, and its structure and the mechanism of its DNA replication have been extensively studied (5, 6). ɸ29 forms tiny plagues on lawns of B. subtilis 168 even though it productively infects this bacterium in liquid medium (7). However, the molecular mechanism by which DynA interferes with phage infection remained unclear.

Recently, a variety of new bacterial resistance mechanisms against phage infection have been discovered. Phage infection imposes a tremendous pressure on bacteria to develop viral resistance strategies for survival (8–10). The evolution of bacterial phage defense systems in turn promotes the evolution of anti-resistance systems in phages and consequently, phages rapidly coevolve to bypass bacterial antiviral systems (11). Bacteria have evolved mechanistically diverse defense strategies that act at almost every stage of the phage infectious cycle. A first line of defense for bacteria is blocking phage attachment to the specific receptors, e.g., via production of extracellular matrix to occlude receptors, or via exploitation of competitive receptor inhibitors (12–19). Production of extracellular vesicles has also been proposed to act as a decoy mechanism to prevent phage attachment to cells (20). Also, block of phage DNA injection can be achieved by superinfection exclusion proteins that are commonly phage or prophage encoded (21, 22). However, the most abundant strategy to prevent phage infection is the specific degradation of viral nucleic acids by bacterial restriction modification systems, or by CRISPR/Cas systems that enable protection from invading DNA (23–27). While these mechanisms are acting on a single cell level and protect the individual cells, there are also systems acting on the population level. One of these mechanisms is abortive infection, which inhibits phage assembly in the infected cells by host cell lysis before mature viral particles have been assembled, thereby efficiently protecting the distribution of phages in the bacterial population (28, 29). Abortive infection, and thus early sacrifice of the infected cells, is among the most effective protection mechanism against rapid spreading of virus infections.

Here, we describe a novel phage resistance mechanism that prevents efficient host cell lysis. B. subtilis cells expressing the bacterial DLP DynA lyse later and with lesser efficiency compared to cells lacking this bacterial DLP. Single-cell infection and phage replication are not affected by DynA. Thus, DynA provides a protective effect only on the population level. Upon phage infection, DynA forms large, confined clusters at the cell membrane, likely preventing membrane rupture. Our data reveal that bacterial DLPs act as the first reported bacterial anti-phage system that blocks phage release from infected cells. DLPs are widespread in bacterial genomes and their combined activity against pore forming antimicrobials and their protective effect against virus dispersal in bacterial populations make them important parts of the bacterial stress response and innate immunity systems.

## RESULTS

### DynA provides protection against phage infection.

**FIG 1:**
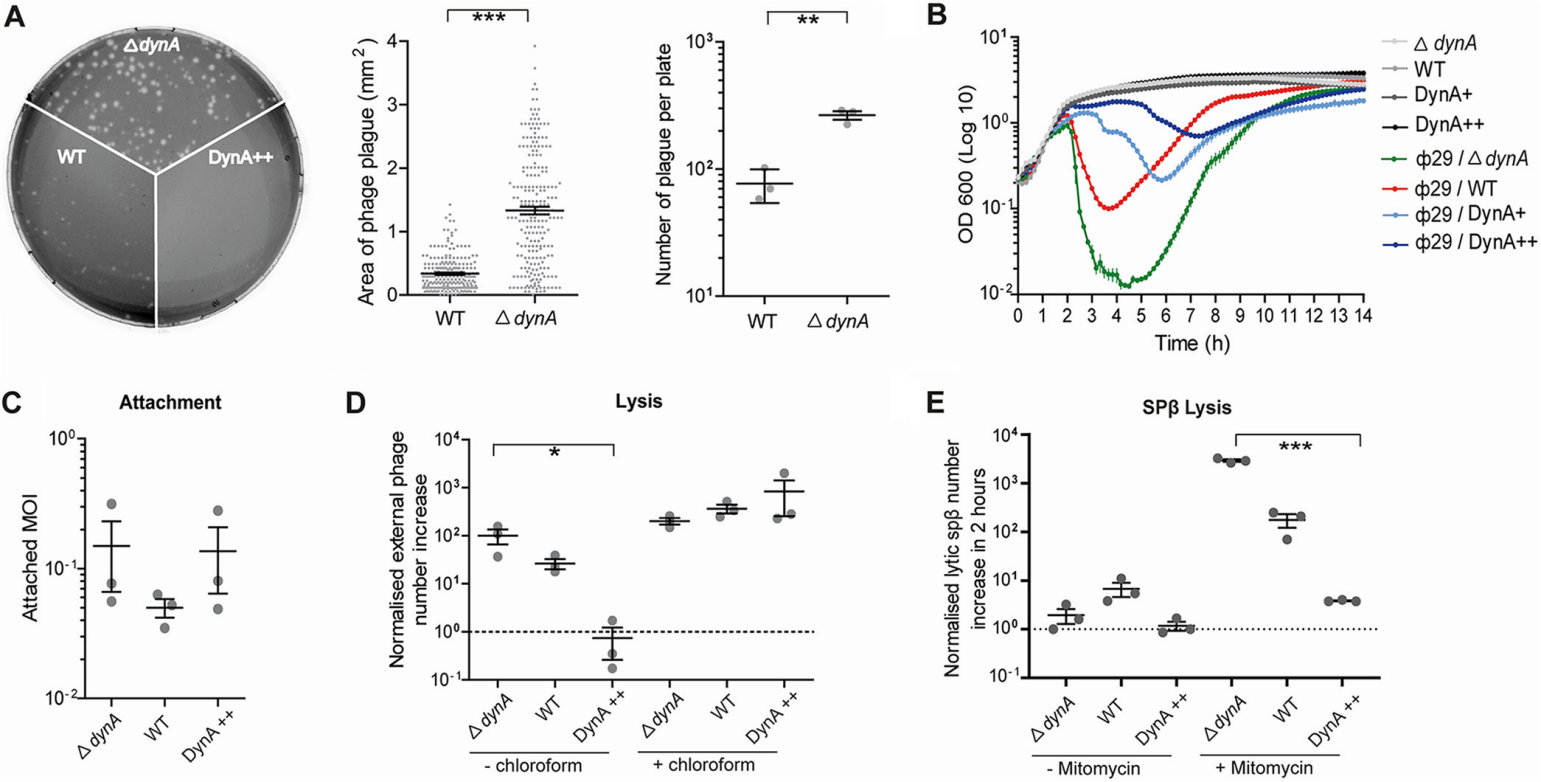
DynA confers resistance against phage infection. (A) ɸ29 plaque formation on wild type B. subtilis 168 (WT), DynA-deficient strain (Δ*dynA*) and cells overexpressing DynA (DynA++). Note that almost no plaques can be observed when DynA is overproduced. Quantitative analysis of plaque sizes revealed significant larger plaques in Δ*dynA* compared to wild type. ɸ29 plaque formation causes more plaques in Δ*dynA* cells compared to wild type. (B) The lytic effect of ɸ29 infection (MOI = 1) can be suppressed by DynA overexpression. Optical densities of liquid growth cultures were measured in a plate reader. DynA+ was previously induced with 1% xylose for 30 min, but the xylose not present in growth experiment. For higher DynA expression levels 1% xylose was present throughout the infection experiment (DynA++). (C) Phage attachment in a mixture of bacteria and ɸ29 (MOI = 1) was placed at 24°C for 10 min to allow phage attachment and then cells were separated from extracellular phages. Attachment rates were calculated based on quantitative spot assays. Note that there is no significant difference in phage attachment rates for wild type, Δ*dynA*, or DynA overexpression (DynA++) strains. (D) DynA overexpression reduces the number of released phages after infection. Phages were quantified by spot assays. Release of phages in the supernatant after infection (sample without chloroform) indicates a significant reduction of released phage progeny in cells overexpressing DynA. The total number of replicated phages (inside host cells and released phages) was quantified after chloroform treatment. Note that no difference in total phage numbers in wild type, Δ*dynA*, or DynA overexpression (DynA++) strains is observed. (E) In cells lacking DynA (Δ*dynA*) the lytic induction of the lysogenic phage SPβ is significantly higher than wild type after 2 h mitomycin treatment. Overexpression of DynA (DynA++) drastically reduces SPβ plaque formation. Statistical analysis is based on Student's *t* test (*, *P* value less than 0.05; two (**) less than 0.01; three (***) less than 0.001). Mean and standard error of three replicates are shown.

We have shown before that B. subtilis cells lacking DynA are more sensitive to phage infection by ɸ29 and SPβ (3). We now aimed to unravel at which state of infection this protective effect is exerted. Therefore, we used the lytic B. subtilis phage ɸ29 for infection assays. In line with our earlier observations we found that infection of B. subtilis with ɸ29 causes small plaques. Infection of a *dynA* knockout strain (strain FBB002), however, results in a significant increase in plaque size (Δ*dynA*, *P* = 1.652 × 10^−46^) (Fig. 1A). Furthermore, the number of plaques was increased about four times when a lawn of *dynA* cells was infected compared to that of wild-type B. subtilis *168* (*P* = 0.0014) (Fig. 1A), indicating that the lack of DynA renders cells more susceptible to lysis upon phage infection. Importantly, a strain in which we overproduced a DynA (here a functional DynA-GFP fusion in a strain background where the original *dynA* gene was deleted, strain FBB018) from an ectopic locus under the control of a xylose inducible promoter (DynA++) did not produce any plaques after infection (Fig. 1A) indicating that an increased DynA level leads to a higher resistance. The observable effect of DynA on plaque formation becomes even more obvious with increasing cultivation time. The difference in the amount of phage induced plaques increased up to 25 h of incubation between WT and Δ*dynA* cells (Fig. S1A). After 25 h of incubation, resistant bacteria started to form visible colonies and populated the plates again in wild type and Δ*dynA* cells, indicating that these cells acquired a resistance (Fig. S1A). We also analyzed the lysis behavior induced by ɸ29 in liquid cultures. Exponentially growing cultures were infected with an MOI (multiplicity of infection) of 1 and optical densities were measured. Lysis of DynA-deficient bacteria was significantly faster than that of the wild-type cells (Fig. 1B). We also tested the effect of DynA overexpression. Therefore, we used a strain (FBB018) that expresses an ectopic copy of DynA (a DynA-GFP fusion). Cells were precultured with 1% xylose to induce DynA production, but xylose was removed during the infection experiment (DynA+). These cells lysed much later compared to the wild-type cells and the optical density did not lower as much (Fig. 1B), indicating that the DynA proteins provided a protective effect. In order to increase the DynA amount further we also performed the infection assay with 1% xylose present throughout the infection (DynA++). The continuous overexpression of DynA reduced host cell lysis after ɸ29 infection drastically (Fig. 1B). We concluded from this experiment that DynA has a protective effect but may not entirely prohibit phage infection of cells. We have shown before that DynA is a GTPase and GTP hydrolysis accelerates efficient fusion of membrane vesicles *in vitro* (content mixing) (2), while GTP-binding and hydrolysis was not required for membrane tethering and lipid mixing. Therefore, we tested whether GTP hydrolysis of DynA is required for the observed effect on phage infection in B. subtilis. To this end we used a strain expressing a DynA mutant strain in which both signature lysine residues K56 and K625 were changed to alanine residues (K56A/K625A variant, strain LJB013). The mutated DynA variant was expressed as a Dendra2 fusion as the only DynA copy ectopically from the *amyE* locus under the control of a xylose inducible promoter. We compared the infection of the K56A/K625A variant in growth assays with 1% xylose present throughout the infection (MutDynA++) with the overexpression of the wild-type protein (DynA++). When only the mutated protein was present in the cells, no protective effect was observed, and cells lysed to a similar degree as the null mutant (Fig. S2). We conclude that GTP-binding and hydrolysis is essential for the protective effect of DynA during infection with phages. DynA is a membrane associated protein, and we have shown before that it is able to modulate membrane fluidity (3). Overexpression of membrane associated proteins could therefore have an indirect effect on phage infection any the effects observed with DynA could be unspecific. To control for this, we used strains in which we overexpressed the membrane associated protein MinD and DivIVA (strains BB009 and JBB018, respectively) (30, 31). Both constructs are fusions to Dendra2 and in these strains the native *dynA* expression was still intact. Overexpression of MinD and DivIVA was again induced by addition of 1% xylose. Growth curves after infection revealed that MinD or DivIVA overexpression have only a mild effect on cell lysis (Fig. S3A). Thus, overexpression of membrane associated proteins can to some extent influence phage infection, however, the effect of DynA on phage infection is clearly stronger. The specific effect of DynA toward infection becomes even clearer when we compared plaque assay formation of cells overexpressing DynA, MinD and DivIVA (Fig. S3B). While overexpression of MinD and DivIVA does not reduce plaque formation, overexpression of DynA almost entirely abolishes plaque formation. Thus, DynA exerts a specific effect on phage infection.

10.1128/mbio.03753-21.1 FIG S1 (A) Plaque formation of wild type and Δ*dynA* strains after infection with ɸ29 phage. Plates without phage infection are shown as control. Plates were incubated at room temperature and imaged for 38 h. Quantification of plaque formation was analyzed by gray values based on the pate images. (B) The proportion of bacteria attached to the phages after 10 min at 24°C in experiments with various amounts of phages (MOI 1000, 10 and 1). Download FIG S1, TIF file, 2.9 MB. Copyright © 2022 Guo et al. 2022 Guo et al. https://creativecommons.org/licenses/by/4.0/ This content is distributed under the terms of the Creative Commons Attribution 4.0 International license.

10.1128/mbio.03753-21.2 FIG S2 Overexpression of a catalytically inactive DynA variant (K56A, K625A mutant, strain LJB013, expressing DynA-K56A, K625A-Dendra2, Mut.DynA++) does not provide population resistance against phage infection. The lytic effect of ɸ29 infection (MOI = 1) in liquid cultures in cells expressing the mutated DynA (Mut.DynA++) and the DynA-GFP fusion protein (DynA++) is compared to strains lacking the *dynA* gene (Δ*dynA*) and wild type (WT). Optical densities of liquid growth cultures were measured in a plate reader. Overexpression of DynA and the mutated DynA was induced with 1% xylose during precultures and throughout the infection experiment. Download FIG S2, TIF file, 0.2 MB. Copyright © 2022 Guo et al. 2022 Guo et al. https://creativecommons.org/licenses/by/4.0/ This content is distributed under the terms of the Creative Commons Attribution 4.0 International license.

10.1128/mbio.03753-21.3 FIG S3 Overexpression of DivIVA and MinD has little effect on phage infection of B. subtilis. (A) The lytic effect of ɸ29 infection (MOI = 1) is only mildly affected by DivIVA or MinD overexpression in liquid cultures. Optical densities of liquid growth cultures were measured in a plate reader. Overexpression of MinD or DivIVA was induced with 1% xylose during precultures and throughout the infection experiment (DivIVA++, MinD++). (B) Plaque formation of cells overexpressing DivIVA (DivIVA++), MinD (MinD++), or DynA (DynA++) in comparison to wild type (WT168) after infection with ɸ29 phage. Note that neither DivIVA nor MinD reduces the number of plaques (light spots) compared to the situation in wild type, while DynA overexpression almost renders the culture resistant to phage infection. Download FIG S3, TIF file, 0.2 MB. Copyright © 2022 Guo et al. 2022 Guo et al. https://creativecommons.org/licenses/by/4.0/ This content is distributed under the terms of the Creative Commons Attribution 4.0 International license.

### DynA provides protective effects against phages and prophages.

We next tested phage attachment and host cell lysis during the first infectious cycle for the three strains [wild type (168), Δ*dynA* (FBB002), and DynA++ (strain FBB018)] (Fig. 1C). A mixture of bacteria and ɸ29 (MOI = 1) was placed at 24°C for 10 min to allow phage attachment. Subsequently, cells and attached phages were separated from free phages and the titer of free phages was tested by quantitative spot assays. Phage attachment was similar in all three strains (*P* = 0.3005 for Δ*dynA*: WT; *P* = 0.9129 for Δ*dynA:* DynA++). The average attachment rate of ɸ29 was around 10%. When the MOI was higher than 10, phages were attached to all cells (Fig. S1B). These results indicate that DynA does not significantly influence phage attachment. Next, we tested phage assembly and host cell lysis using quantitative spot assays (Fig. 1D). To this end, cells were mixed with phage ɸ29 at an MOI of 1.0 and incubated at 37°C for 1 h. Then, cells were harvested, and the bacterial cell membrane was destroyed by chloroform treatment, thereby releasing the fully assembled phage progeny. This method reliably lyses all bacterial cells independent of DynA concentrations, allowing us to compare a potential effect of DynA on intracellular phage assembly. After 1 h of infection, the number of released phages and fully assembled phages was measured using quantitative spot assays. As a control we also determined the number of free phages that have been released independent of the chloroform treatment. In experiments without chloroform lysis of the host cells, the increase in active phages was highest for cells lacking DynA. However, the difference between Δ*dynA* and wild type was not significant for the three biological replicates we made. In contrast, in a strain overexpressing DynA (DynA++, strain FBB018) we did not observe an increase in phage numbers during the infection and the difference in released phages between Δ*dynA* and an overexpression strain (DynA++) was significant (*P* = 0.0453 for Δ*dynA:* DynA++). When cells were lysed by chloroform correctly assembled phage particles that were not naturally released by host cell lysis were also liberated and thus the chloroform treatment reveals the total number of assembled phages. We observed no difference of phage numbers between wild type, Δ*dynA*, and DynA++ cells, indicating that DynA does not alter the ɸ29 infection rate, or phage replication and assembly, but rather the release of mature phage particles.

The B. subtilis 168 strain we use for our experiments carries the SPβ prophage integrated into its genome. Upon cellular stress, such as DNA damage, SPβ is activated and assembled, leading to cell lysis (7). We were wondering whether the bacterial DLP DynA might only act against external, lytic phages or whether it may also have an effect on prophage proliferation. To address this idea, we performed a phage lysis test by inducing the lytic cycle of prophage SPβ by mitomycin C addition (Fig. 1E). The release of SPβ phage particles was tested by plating the supernatant from infection assays on a lawn of a SPβ-free B. subtilis strain 25152, lacking (PSB026) the *dynA* gene. Induction of SPβ in wild type 168 was observed and a low frequency of plaques was observed. However, when cells were treated with mitomycin C, the number of plaques increased significantly (*P* = 0.0408 for WT:WT with mitomycin) (Fig. 1E). B. subtilis cells lacking *dynA* (strain FBB002) released a similarly small number of phages compared to the wild type when cells were not stressed by mitomycin C. However, when cells were treated with the DNA damaging agent mitomycin C, a large number of plaques were observed on a lawn of the susceptible PSB026 cells (Fig. 1E). Therefore, absence of DynA resulted in a significant (*P* = 0.0005 for Δ*dynA* with mitomycin:WT with mitomycin) increase of SPβ production and release. Importantly, when we overproduced DynA (strain FBB018), we observed almost no increase in plaque formation after SPβ induction, indicating that an increase of DynA concentration significantly decreases the SPβ release (*P* = 8.8133 10^−5^ for Δ*dynA* with mitomycin:DynA++ with mitomycin) (Fig. 1E). Therefore, we conclude that the protective effect of DynA does not specifically act on ɸ29, but seems to be a general mechanism interfering with viral release.

### DynA is not prohibiting phage replication in single cells.

**FIG 2:**
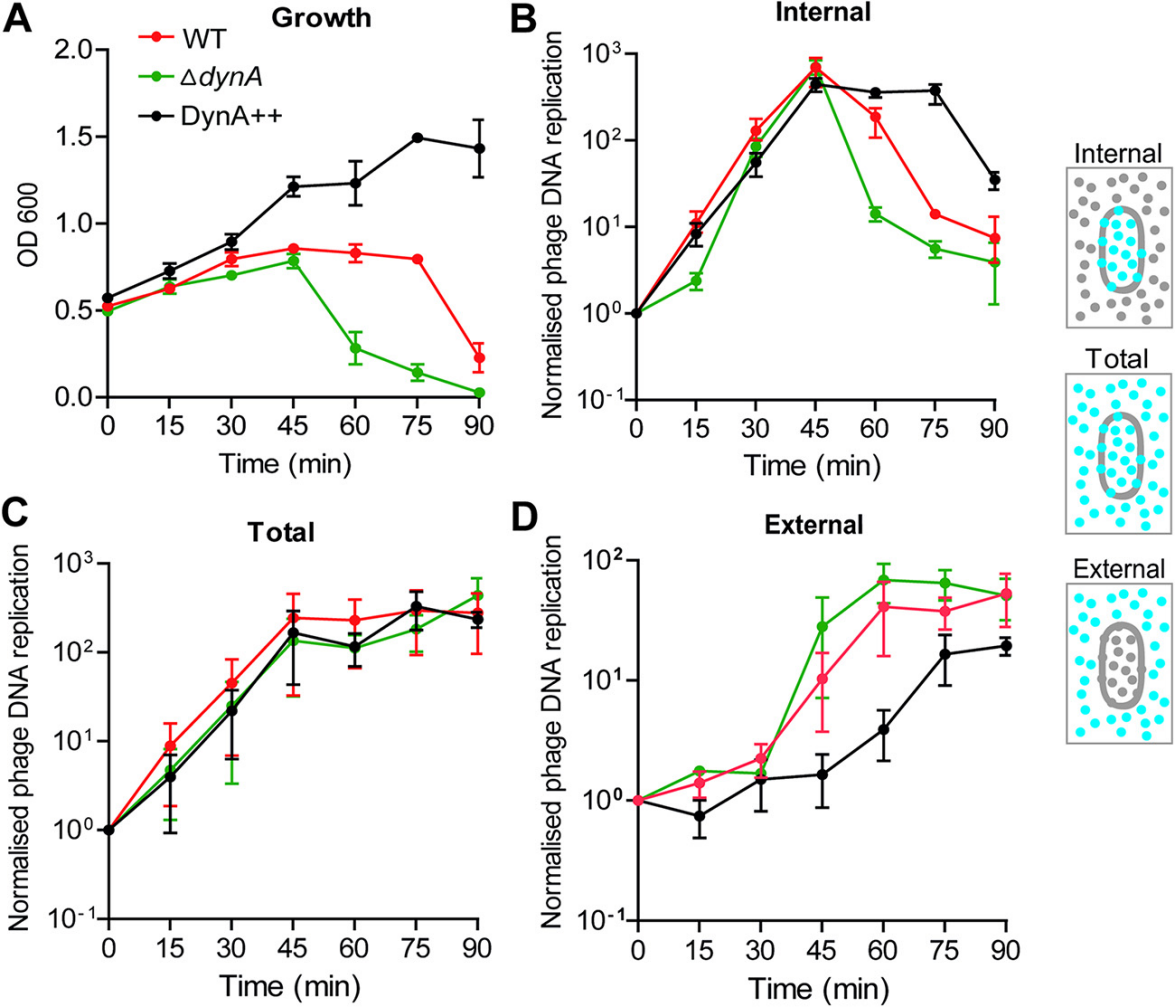
Phage DNA replication analyzed by qPCR. Phage DNA was quantified in B. subtilis wild type, Δ*dynA*, and the overexpression strain (DynA++) after ɸ29 (MOI = 10) infection. The mixture of bacteria and ɸ29 was preplaced at 24°C for 10 min to allow phage attachment and then transferred to shaker of 37°C for measurement. Phage DNA of inside or on the cell, in total, and outside the cell were quantified every 15 min (see Materials and Methods). (A) Optical density of infected cultures measured at 600 nm. Note the absence of a strong lysis effect in the DynA overproduction strain after phage infection. (B) Quantification of intracellular phage DNA. After 45 min the DynA overexpression strain contains more phage DNA trapped inside the host cell compared to the wild type and the Δ*dynA* strain. (C) The total phage DNA content in the samples is highly similar in all three experimental condition. (D) Quantification of external (released) phage DNA reveals that in cells overexpressing DynA less phages are released over the course of the experiment. Mean and standard error of three replicates are shown in all experiments.

In order to precisely determine whether DynA only affects viral release and not phage replication or phage assembly, we used qPCR to quantify viral DNA in wild-type cells (168), Δ*dynA* (FBB002) and a DynA overexpression strain (FBB018) individually for internal, external and total phage DNA (Fig. 2). To test for the effectivity of phage infection we monitored again cell lysis in liquid cultures by measuring optical densities (Fig. 2A). When the MOI was equal to 10, the cell number of the Δ*dynA* strain dropped significantly faster compared to wild type around at 45 min postinfection. In contrast a strain overexpressing DynA (FBB018) continued growing and showed no sign of massive cell lysis. Before 45 min of incubation, there was no significant difference in the growth rates between the three strains. We then analyzed the intracellular concentration of viral DNA (see Materials and Methods) between the three strains. Within the first 45 min after infection the increase of viral DNA was similar in all three strains (*P* = 0.5189 for Δ*dynA:* DynA++; *P* = 0.9382 for Δ*dynA:* WT) indicating that DynA did not affect the replication of phage DNA. After 90 min the concentration of internal viral DNA was higher in the cells overexpressing DynA and lowest in cells lacking the bacterial DLP (Fig. 2B). To test whether the total phage production was similar in all strains, we quantified the total phage DNA and found that it remained indistinguishable within 90 min between all three strains (*P* = 0.9821 for Δ*dynA:* DynA++; *P* = 0.6218 for Δ*dynA:* WT) (Fig. 2C). This argues that phage replication (and hence viral DNA production) is not affected by DynA, but that cells lacking DynA release the viral DNA earlier into the medium, while cells with increased DynA concentrations seem to lyse slower and less efficient. If total phage production is similar, but cells lacking DynA lyse faster, we should find more viral DNA outside the cells in the medium. We therefore separated cells from medium and determined the concentration of viral DNA that has been released. In line with our hypothesis, we found more external viral DNA in Δ*dynA* cells and the lowest concentration in cells overexpressing DynA (Fig. 2D). Together, these data indicate that Δ*dynA* cells released the phage DNA approximately 45 min after infection more efficiently, while the lysis process of wild-type and overexpression strains were delayed. In summary, the presence of DynA did not influence phage attachment or viral DNA replication and phage assembly, but significantly slowed down host cell lysis and phage dispersal.

### DynA does not colocalize with attached phages or viral DNA entry sites.

**FIG 3:**
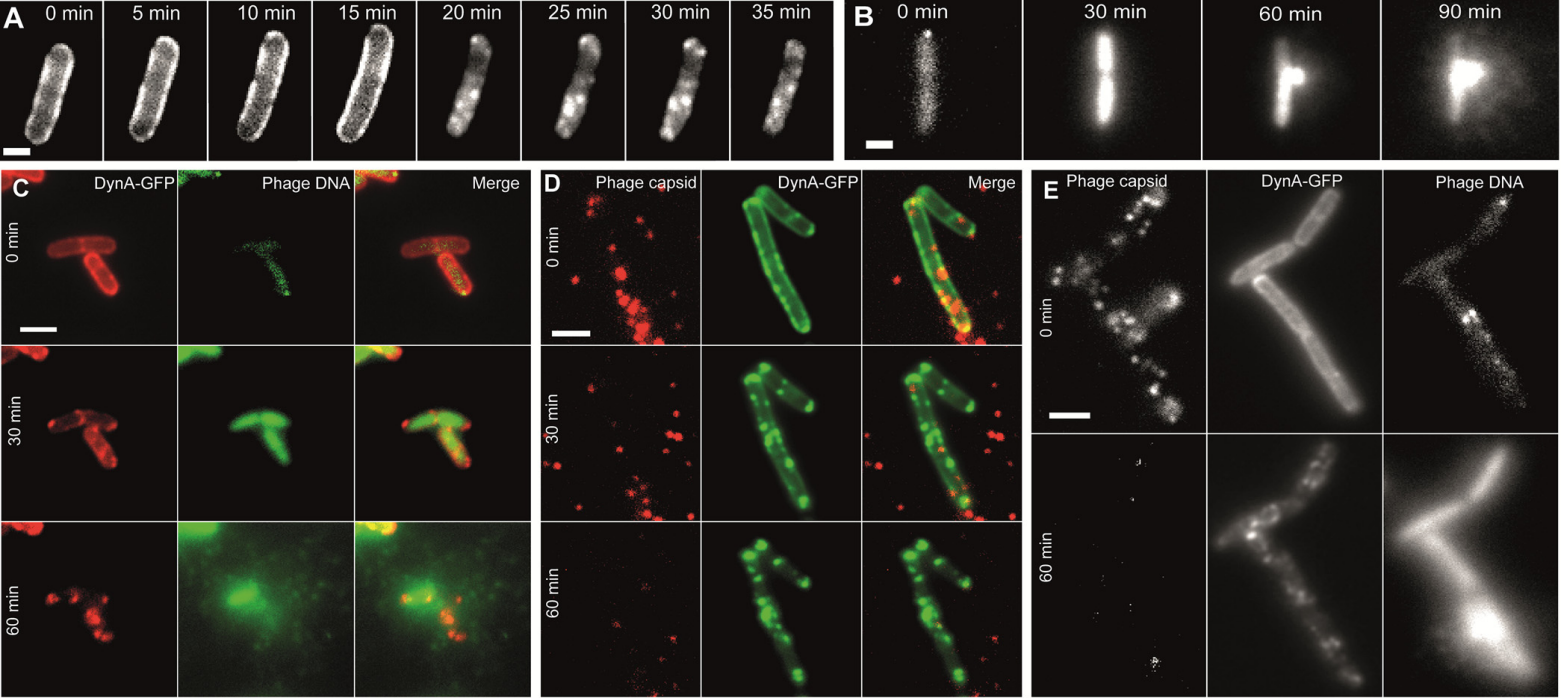
The *in vivo* dynamics of DynA during ɸ29 infection observed by fluorescent labeling. (A) ɸ29 can induce DynA oligomerization *in vivo*. When the bacteria were exposed to phages (MOI= 1), DynA assembles into large clusters approximately 20 min after phage attachment at 37°C. Scale bar 1 μm. (B) Phage infection leads to host cell lysis. Phage DNA labeled with Hoechst DNA stain was used to track the infection progression. Upon attachment of the DNA-labeled phage (0 min) a small fluorescent focus can be observed on host cells. 30 min after infection the DNA stain has been injected into the host cell along the viral DNA. 60 min after infection cell lysis starts with bulging of cytoplasmic content, including the fluorescently stained DNA. 90 min after infection the membrane bursts and DNA, likely, including phage particles, is released. Scale bar 1 μm. (C) Analysis of DynA-GFP (false colored red) dynamics in infected cells. Phage infection is monitored by Hoechst dye injection into host cell by DNA-labeled phages (false colored green). Scale bar 2 μm. (D) Colocalization of ɸ29 phages with labeled capsids (Alexa Fluor 647 dye, red) and DynA-GFP (green). Note that there is no correlation between localization of the phages and DynA at the onset of the infection cycle. Scale bar 2 μm. (E) Infection of cells expressing DynA-GFP (green channel) with double labeled phages (DNA stained with Hoechst [blue channel] and capsids labeled with Alexa Fluor 647 [far-red channel]). Scale bar 2 μm.

We next wanted to visually follow the viral infection process and host cell lysis. Therefore, we fluorescently labeled phages and DynA *in vivo* to study the interaction between them on a single-cell level. To follow DynA dynamics inside the cell we used a DynA-GFP fusion protein expressed ectopically (from an *amyE* locus) under the control of a xylose inducible promoter. The endogenous *dynA* gene in this strain was deleted (strain FBB018) (1). As described before, in exponentially growing cells, DynA localizes around the cell periphery, associated with the plasma membrane. Addition of ɸ29 phage lead to a change of DynA localization. Approximately 20 min after phage addition, but not before, DynA started to form clusters along the cell membrane (Fig. 3A). Thus, cluster formation did not occur during the early stages of phage infection, and hence, DynA clustering is likely not induced by phage attachment or DNA injection. The observed cluster formation of DynA was similar to the effects that we described before when cells were treated with nisin (3). We next labeled the phage DNA with the DNA-specific dye Hoechst. When we mixed phages with cells, we were able to visualize quickly cells that showed a small, bright blue fluorescent focus. During the course of infection, this focus disappeared and instead the entire cytoplasm of the infected cell was stained with Hoechst, indicating that the DNA dye is now dispersed throughout the infected cytoplasm. Since the bacterial nucleoid is degraded during virus replication (32), we assume that the majority of the stained DNA corresponds to viral DNA that is replicating in the host (Fig. 3B). The infected cell eventually lyses and the DNA bulges out of the host cell, which finally bursts and fragments, indicating dispersal of phage progeny (Fig. 3B). When we simultaneously followed DynA-GFP, and the cells were mixed with the DNA-labeled phage, we were able to observe that DynA aggregation occurred before host cells burst, releasing phage particles (Fig. 3C). We then wanted to label phage capsids and phage DNA simultaneously. To this end, we mixed CsCl-purified phages with Alexa Fluor 647 succinimidyl-ester (see Materials and Methods). The succinimidyl-ester covalently attaches the dye to free amino groups of capsid lysine residues. These labeled phages retained their full infection potential and readily infected control cells. We used these capsid labeled phages for infection experiments using the strain expressing the DynA-GFP fusion protein (strain FBB018). Directly after mixing phages and cells, we observed a large number of phage particles close to the cell surface of the bacteria. However, DynA remained evenly distributed along the plasma membrane. During the course of the infection, the phage capsids gradually detached from the cells over time or decayed, leading to a disappearance of the far-red Alexa-647 signal (Fig. 3D). DynA clustering did not coincide with phage capsid localization during the early stages of infection, ruling out that DynA would react to phage attachment or viral genome injection. Furthermore, the images show that DynA did not colocalize with the attached phage capsids, as indicated by the absence of an overlap of green and red fluorescence. Finally, we tracked localization of viral capsids, viral DNA and DynA simultaneously. Therefore, we used the Alexa-647 labeled phages and stained their DNA with Hoechst. Also, the double labeled phages retained their full infection potential. Hoechst dyes are cell permeable and could potentially leak out of the labeled phages and strain bacterial cells that are not infected. We controlled for this by mixing labeled phages with Escherichia coli cells. E. coli is not infected by Φ29 and hence, any Hoechst fluorescence in these cells would stem from dye leakage. We did not observe any increase in intracellular fluorescence if E. coli cells when mixed with labeled phages (Fig. S4) within the time course of an infection assay (90 min). When we infected DynA-GFP expressing cells, we saw that also at those sites where DNA was injected into the host cells (visualized by bright blue fluorescent foci), DynA did not accumulate. However, during later stages of the infection cycle DynA formed clusters that were enriched in the vicinity of phage egress sites (Fig. 3E). Taken together, these experiments argue for an involvement of DynA at sites in the cell membrane during release of the phage progeny. Missing colocalization of DynA with attaching phages and viral DNA injection, as well as the phage titer experiments, make it unlikely that DynA plays a role in prevention of infection at the single cell level.

10.1128/mbio.03753-21.4 FIG S4 Hoechst labeled phages do not leak dye during experiments. In order to control for a putative leakage of the hydrophobic Hoechst dye from labeled phages, we mixed the labeled and PEG purified phi29 phages with Escherichia coli DH5alpha cells and imaged (exposure time 70 ms) for Hoechst fluorescence. Samples were taken every 30 min from a shaking culture. No fluorescence increase in the E. coli cells was observed over the time scale that was used in infection assays. Note that the phage particles are visible as small, diffraction limited foci. We conclude that the phage capsid efficiently retains the Hoechst dye in the phages. Download FIG S4, TIF file, 0.3 MB. Copyright © 2022 Guo et al. 2022 Guo et al. https://creativecommons.org/licenses/by/4.0/ This content is distributed under the terms of the Creative Commons Attribution 4.0 International license.

### DynA delays and reduces host cell lysis.

**FIG 4:**
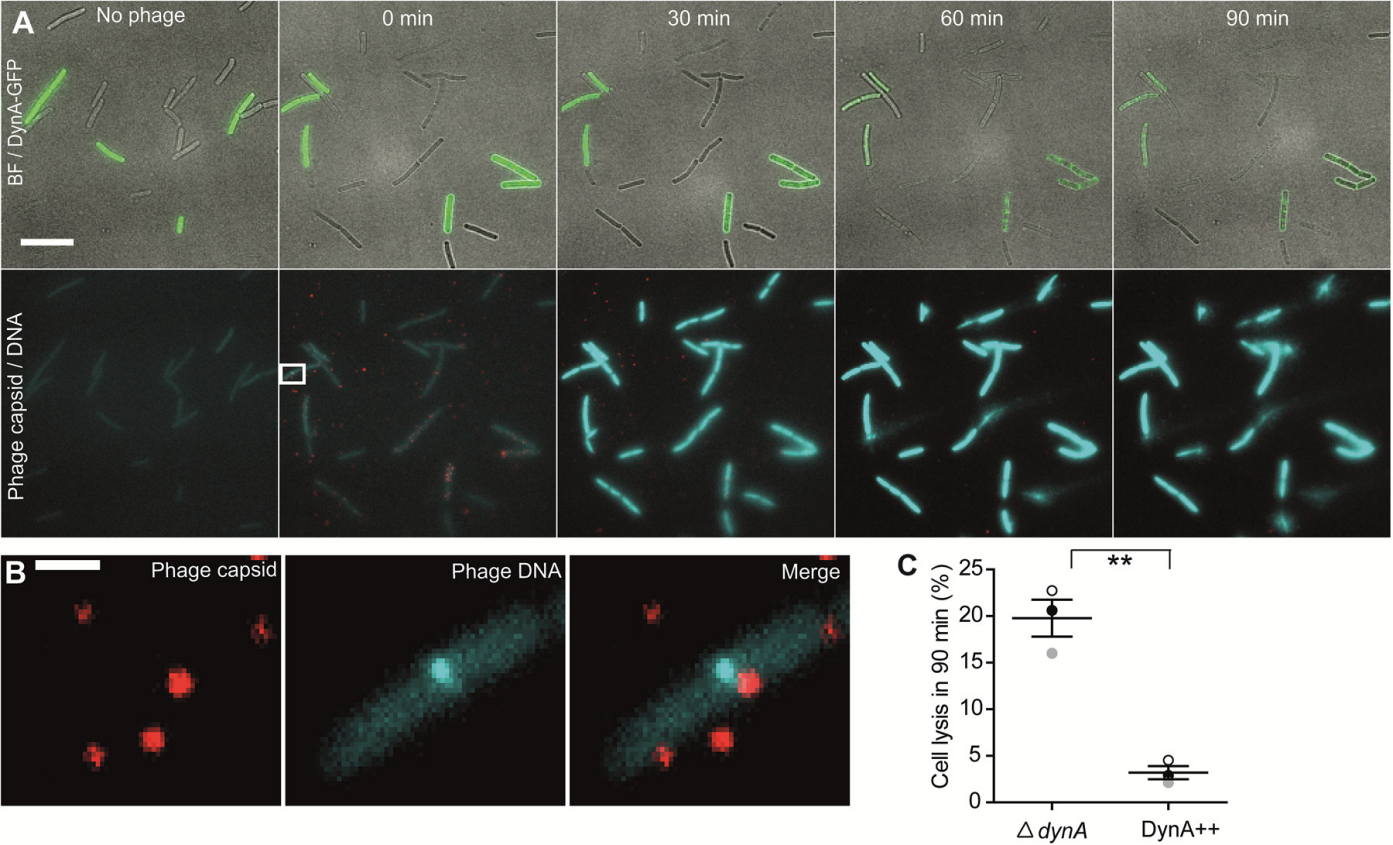
DynA prevents host cell lysis after infection. (A) Fluorescent microscopy analysis of a mixed culture of a DynA-overexpressing strain (DynA-GFP induced by 1% xylose, cells expressing DynA are indicated by green fluorescence) and a DynA-deficient (Δ*dynA*) B. subtilis strain after infection with ɸ29 (phage DNA labeled with Hoechst dye and capsids labeled with Alexa Fluor 647). Time lapse analysis of the infection process reveals that shortly after addition of the labeled phages red foci appear next to the host cells and in few cases bright blue foci inside the host cells can be observed. Cells that are actively infected can be identified by the intracellular Hoechst DNA stain (blue). Cells with and without DynA are infected equally well. During the time course of the infection more of the cells lacking DynA lyse while cells expressing DynA remain intact. (B) Zoom into an early infection where the Hoechst dye injection into the host cell is spatially close to a labeled capsid, indicating an ongoing infection process. Note that no DynA focus is present at the site of DNA injection Scale bar 1 μm. (C) Quantification of bacterial lysis of DynA overexpressing (DynA++) and the deletion (Δ*dynA*) strains.

The results indicated that the protective effect of DynA on phage infection is unlikely happening at the stages of phage attachment and viral DNA replication. Thus, we reasoned that DynA may have an effect on host cell lysis and probably phage release. This would be in line with the biochemical activity of DynA in membrane protection by tethering and fusion reactions (1, 2). However, the extent of host cell lysis is difficult to quantify in different infection experiments, since there is a large variation in the degree of host cell lysis. To circumvent this problem, we designed an experiment in which cells deleted for *dynA* and cells expressing DynA-GFP were mixed. GFP fluorescence identified the cells that contain DynA, while cells without GFP fluorescence were Δ*dynA* cells. Fluorescence microscopy of the mixed cell cultures revealed that cells with and without DynA can be identified easily, while we did not observe fluorescence signals in the blue and far-red channel (Fig. 4A). We than added the phages that we labeled on their capsids with Alexa-647 and the viral DNA with Hoechst dye. Immediately after mixing (0 min) we readily observed the far-red labeled virus capsids and, in few occasions, also small bright blue foci inside the B. subtilis cells (Fig. 4A, white square). A magnification of the cell shows that the Hoechst label is directly underneath an Alexa-647 signal, indicating that this is likely an event in which the phage DNA is injected into the host cell (Fig. 4B). The bulk of the bacterial nucleoid remained unstained at this stage of infection. After 30 min, most cells showed a dispersed Hoechst signal, indicating that by now most bacteria were infected and the Hoechst dye entered the host cells with the viral DNA. This allows an easy quantification of those cells that are infected. Together with the DynA-GFP signal we can quantify whether there is an infection bias between Δ*dynA* cells of DynA-GFP expressing cells. This was not the case, and thus, both strains were infected with the same rate. 60 min after infection we observed that the first cells lysed; indicative for lysis was the dispersal of the blue Hoechst dye diffusion. 90 min after infection many cells that lacked DynA (Δ*dynA*) were lysed, but most cells that contained DynA (DynA-GFP) were still intact and not lysed, yet (Fig. 4A). Finally, we compared the cell lysis ratio of Δ*dynA* cells and cells expressing DynA-GFP (Fig. 4C). We found that the ratio of cell lysis in Δ*dynA* was significantly higher than the cells expressing DynA-GFP measured 90 min postinfection (Fig. 4C) (*P* = 0.0014 for Δ*dynA*: DynA++). These data clearly speak for a role of DynA in prevention of host cell lysis upon viral replication.

### Single-molecule tracking analysis reveals DynA cluster formation upon phage infection.

**FIG 5:**
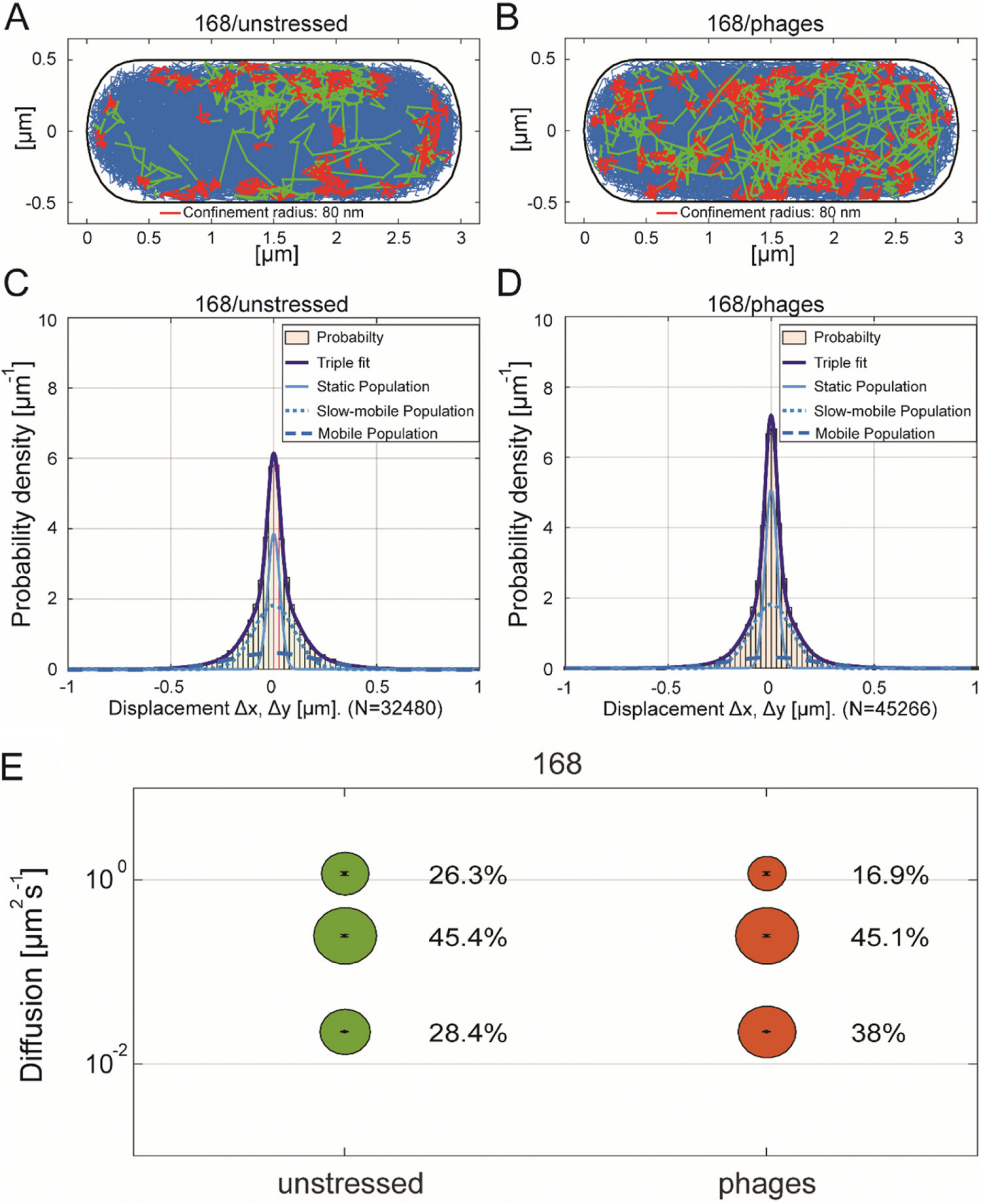
Changes of DynA dynamics in response to phage infection analyzed by single molecule tracking. Tracks of DynA-mVenus displayed in a standardized cell of 1 × 3 μm in (A) unstressed exponential growing cells or in (B) cells infected with ɸ29 bacteriophages after 60 min. Freely diffusive tracks are shown in blue, confined tracks within a radius of 80 nm and a step length of 9 are depicted in red, and transient tracks showing mixed behavior are shown in green. (C–E) Diffusive behavior of DynA-mVenus in Gaussian-Mixture-Model (GMM) analyses of frame to frame displacements in *x*-and *y*-directions in exponential growth phase (C) unstressed or (D) stressed with ɸ29 bacteriophages. The dark blue line indicates the overall fit of the three Gaussians distributions. Dashed, dotted and solid lines in brighter blue are the Gaussian distributions corresponding to mobile, slow mobile and static fractions, respectively. (E) The diffusion constants [μs^2^s^−1^] fraction sizes ([%] and bubble size) are shown in comparison to the two conditions of DynA-mVenus with a step size distribution of three populations, a static (lower bubbles), an intermediate mobile (middle bubbles) and a fast mobile (upper bubbles) fraction.

We also wished to bolster the findings of an increased formation of DynA foci at the cell membrane using single-molecule tracking (SMT). In addition to statically positioned molecules visible by epifluorescence, SMT also visualizes and quantifies freely diffusing molecules. We used SMTracker software to analyze tracking data (33). If DynA was to diffuse throughout the cells, and would become more engaged in the repair of membrane irregularities in response to phage infection, we would expect a decrease of freely diffusing molecules and an increase in statically positioned molecules. We used 20 ms stream acquisition to track DynA-mVenus expressed from the original gene locus, under the control of the original promoter. A projection of all tracks (minimum length of four steps) was plotted into a standardized *Bacillus* cell of 3 × 1 μm size (Fig. 5A). While blue tracks indicate all freely diffusive molecules, red tracks show confined movement of molecules, and green tracks transitions between diffusive and confined movement. Clearly, DynA arrests at the cell membrane in some cases, while predominantly, it is freely diffusive throughout the cytosol. 60 min after infection with phages (MOI = 1), the number of confined, membrane localized tracks strongly increased, as expected (Fig. 5B). In order to further characterize the mode of diffusion of DynA, we employed Gaussian Mixture Modeling (GMM), in which displacements of molecules in x and y direction are evaluated as a probability density function. Tracks with little displacement center around “0,” and fast tracks are away from the central axis. The shape of the function shown in Fig. 5C is clearly not Gaussian, which indicates the existence of at least two populations with different diffusion constants. In fact, data could be best described by assuming three distinct populations. These have diffusion constants of 0.023 μm^2^/s, 0.25 μm^2^/s, and 1.2 μm^2^/s, and sizes of 28, 45 and 26%, respectively (Fig. 5E and Table 1). The populations are most easily explained by assuming a cytosolic, freely diffusive population, most likely consisting of monomers, a membrane-associated fraction diffusing more slowly than cytosolic proteins, and a slow-moving fraction, diffusing in a confined manner, engaged in membrane repair. This fraction has a mobility comparable to translating ribosomes/polysomes (34), and thus appears to have a considerable size. Upon phage infection, the static fraction increases to 38% of DynA molecules, to the expense of the freely diffusive molecules, while the intermediate fraction remained constant (Fig. 5D and E). This observation supports our idea that while more DynA molecules become actively involved in membrane-associated processes, most molecules continue to scan the membrane for lesions.

**TABLE 1:** Diffusion constants of DynA-mVenus

Triple fit	Unstressed	Phages
static D [μm^2^s^−1^]	0.023	0.023
slow-mobile D [μm^2^s^−1^]	0.25	0.25
mobile D [μm^2^s^−1^]	1.2	1.2
static [%]	28.4	38
slow-mobile [%]	45.4	45.1
mobile [%]	26.3	16.9

As a second measure for recruitment into low-mobility membrane clusters, we determined average dwell times of molecules, assuming that catalytically active molecules will remain in a confined motion, and thus statically positioned for many steps. Keeping in mind that dwell times are underestimated in our assays because of molecule bleaching during the acquisition, we can nevertheless conclude that following phage infection, dwell times of DynA strongly increased. During exponential growth, 87.7% molecules had an average, short dwell time of 73 ms (even freely diffusing molecules may stop for a short period of time), and 12.3% of the molecules arrested for 200 ms (Table 2); the latter will largely correspond to the molecules engaged in confined motion at the cell membrane (Fig. 5A). 60 min after infection, 71.3% of DynA molecules showed an even increased dwell time of 290 ms, and only 28.7% of molecules stopped for a short time of 62 ms on average (Table 2 and Fig. 5). Thus, there is a strong shift toward extended dwell times 60 min after phage induction, supporting the idea that DynA molecules react to a strongly increased number of targets within the membrane.

**TABLE 2:** Average dwell times of DynA-mVenus^*a*^

	Exponential	Phages
avg lifetime (frame/s)	7.4/0.15	8.6/0.17
avg residence time τ [s]	0.100 ± 0.011 s	0.283 ± 0.011 s
τ_1_ [s]	0.073 ± 0.00062 s	0.19 ± 0.0062 s
τ_1_ [%]	87.7 ± 1.32 %	28.7 ± 5.23 %
τ_2_ [s]	0.2 ± 0.012 s	0.29 ± 0.0062 s
τ_2_ [%]	12.3 ± 1.32 %	71.3 ± 5.23 %

a τ: dwell time, τ_1_ molecules with a short; τ_2_ molecules with an extended average dwell time.

## DISCUSSION

Bacteria have evolved a multitude of different phage defense systems to survive in a hostile environment where the number of phages exceeds the number of bacteria by an order of magnitude (35). Some of the most successful phage defense systems however, do not protect the individual infected cell, but rather act on a population level. This is the case for all abortive infection systems that lead to host cell lysis before the phage particles have successfully replicated (36, 37). We now describe a novel phage protection system that acts on the population level and exerts its function after phage replication and assembly has been completed. The presence of a bacterial dynamin-like protein, DynA in B. subtilis, interferes with the effective release of phage particles after infection. Thus, similar to abortive infection, dynamin-mediated delay of host cell lysis stops the phage epidemic from spreading rapidly through the population. Although abortive infection is the more powerful mechanism to reduce phage epidemics, the dynamin-mediated phage defense potentially acts as a last line of defense to reduce the speed of the infection process in a bacterial population. Bacterial dynamins are therefore part of the multilayered bacterial response to phage infection with dedicated systems acting at each step of the phage infection cycle. Furthermore, the delay in phage particle release is independent of the phage species. We show here that DynA also exerts its protective effect against activated prophages such as SPβ.

Our data reveal that bacteria lacking DynA are infected at similar rates compared to their wild type siblings. Also, phage attachment and viral replication are unchanged in *dynA* mutant strains. Thus, lack of DynA does not result in an altered cell envelope structure that might reduce phage adsorption, thereby reducing infection rates. Rather, we show that presence of DynA at the cell membrane significantly reduces cell lysis and rupture of the plasma membrane. The protective effect of DynA becomes increasingly obvious, when this protein is overexpressed. It has recently been shown that phage lysis results in explosive cell rupture, but also a blebbing of the membrane with subsequent lysis has been observed (38). We frequently observed infected cells that had large bulges of cytoplasmic material (for example stained by DNA marker Hoechst). Large bulges of cytosolic content that is still surrounded by membrane suggests that these cells do not burst in an explosive and fast manner. We therefore hypothesize that DynA exerts a protective effect on the membrane that leads to a stabilization. This in turn reduces or at least delays the release of viral particles and explains the small plaque phenotype that we observe with cells expressing DynA. Overexpression of DynA enhances this effect drastically and cell lysis is largely reduced. We have shown before that DynA reduces phospholipid dynamics (3) and therefore there is likely a limit for the DynA concentration in the cell that still allows the required membrane fluidity homeostasis. Thus, there will be a tradeoff between fitness and a sufficiently strong protective effect against phage protection. Overexpression of other membrane associated proteins such as MinD and DivIVA have a slight effect on phage infection (Fig. S3) and hence membrane fluidity might be a factor that has some limited effect on viral infections. However, overexpression of MinD or DivIVA do not reduce plaque formation (Fig. S3B). Therefore, overexpression of membrane associated proteins does not affect phage infection in general.

The role of GTP-binding and hydrolysis for bacterial DLPs has long been mysterious. In this study, we show the first clear phenotype of a GTPase deficient DynA mutant. A DynA variant, in which the conserved lysine residues K56 and K625 were mutated, is as sensitive to phage infection as the deletion strain, suggesting that nucleotide hydrolysis is essential for DynA to fulfill its phage defense effect. These data further lend support to the notion that DynA is directly involved in the protective effect during phage infection. It is still speculative which molecular step in the DynA dynamics is regulated by the GTPase switch. We speculated before that GTP hydrolysis is required for the release of DynA complexes from the membrane (1–3). Indeed, localization of the DynA K56A/K625A mutant was shown to be uniform along the membrane and focus formation of this mutant seemed to be affected. Thus, it seems plausible that GTP hydrolysis is required for focus formation and, hence, for formation of the functional DynA complexes that form during membrane stress. Based on data from *in vitro* experiments we hypothesized that GTPase activity is required to release the protein from the membrane, which is essential to keep DynA complexes dynamic and allow response to multiple membrane damages. This is in accord with localization studies and single molecule tracking that clearly show a DynA response to phage infection. While DynA is highly mobile in uninfected cells, the percentage of freely diffusible DynA decrease and the percentage of stationary DynA at the cell membrane increases with infection. We have shown before that DynA is accumulating at sites of membrane damage (3). Thus, likely DynA is recruited to the membrane lesions caused by phage lysis and helps to maintain membrane integrity. Scission and fusion of membranes is a common repair mechanism that is not only known for dynamin related proteins, but also seen with the ESCRT machinery (39). Similarly, it was recently described that the mycobacterial DynA homolog IniA is colocalized with sites of membrane lesion induced by cell envelope acting antibiotics (40). A conserved function of DynA seems therefore the reaction to membrane deformation and rupture. In case of phage induced cell lysis bursting of the membrane is reduced and delayed by DynA. The mechanism of action is therefore quite simple and does not involve sophisticated signal perception. A passive system that just reacts to membrane deformations is ideally suited to provide a last barrier against phage spreading. Also, a simple reduction of the explosive cell rupture will lead to a less effective phage distribution within the colony of bacteria, leading to a reduction of the epidemic spreading.

Dynamins are ubiquitously distributed proteins and they seem to be early inventions in evolution. Stabilizing the plasma membrane against stress is a highly important feature and without dedicated membrane protection systems cellular life is unthinkable. In this aspect membrane protection can be compared to protection of genome integrity. It is therefore surprising how little we know about the molecular mechanisms that are used by cells to protect their cellular integrity. Dynamins seem to be ideally suited to fulfill this role and we should consider them not only as important proteins involved in membrane dynamics, but rather as a membrane protection component that contributes to the bacterial immune strategy.

## MATERIALS AND METHODS

### Strains and media.

Bacterial strains used in this study are listed in Table 3. DynA-mVenus expressed from the original gene locus was constructed by cloning the last 500 bp (excluding the stop codon) of *dynA* into plasmid pSG1164 (41)containing the mVenus gene, using enzymes EcoRI and ApaI, after PCT amplification with forward primer GCTAGAATTCGACAACAGCCTTA and reverse primer GCATGGGCCCCATTTTTATTGTATTGTCTG. B. subtilis 168 was transformed with the resulting plasmid, establishing strain PG2112. The resulting Cells were grown in LB medium (10 g/liter tryptone, 10 g/liter NaCl, 5 g/liter yeast extract) unless otherwise stated. Bacteriophages ɸ29 and SPβ were purchased from the German Collection of Microorganisms (DSMZ GmbH).

**TABLE 3:** Strains used in this study

Strain	Genotype	Reference/source
Bacillus subtilis 168	*trpC2*	Laboratory collection
Bacillus subtilis 25152	*rpC2, metBl O, xin-1, SPβ-(erm)*	Laboratory collection
FBB002	*trpC2, dynA::tet*	(1)
FBB018	*amyE::Pxyl-dynA-gfp spc dynA::tet trpC2*	(1)
PSB026	*trpC2, metBl O, xin-1, SPβ-(erm), dynA::tet*	(3)
LJB013	*amyE::Pxyl-dynA-K56A-K625A-dendra2 spc dynA::tet trpC2*	This study
PG2112	*dynA-mVenus (cm)*	This study
JBB018	*trpC2 amyE::cam Pxyl-minD-dendra2*	Juri Bach
BB009	*trpC2 amyE::cam Pxyl-divIVA-dendra2*	(30)

### Quantitative plague assay and spot assay.

Overnight cultures of B. subtilis inoculated from glycerol stock were 100-fold diluted in fresh LB medium and grown to an OD_600_ of 0.5–1.0. Bacteriophages were diluted with 10-fold serial dilutions (1 to 10^10^) in LB medium. For plague assays, 100 μl of each phage dilution were mixed with 1 mL bacterial culture and the mixture spent 10 min at room temperature. Then with 4 mL LB with 0.5% agar were added to the mixtures and subsequently poured on LB agar plates. For spot assays, 1 mL bacteria were mixed with 4 mL LB complemented with 0.5% agar and poured first, then each phage dilution (5 μL) was dropped to the plate. Plaques were detected after 6 h of incubation at 37°C or overnight incubation (<25 h) at 24°C.

### Phage purification by isopycnic CsCl gradient centrifugation.

1 liter of bacteria-phage mixture was prepared with a phage titer above 10^9^ PFU/mL. Bacteriophages were separated from bacteria by centrifugation at 3,800 g for 10 min. The supernatant contained bacteriophages and smaller cell debris. Phages were further purified by centrifugation at 13,000 g for 2 h. Subsequently, 10 mL gelatin-free SM buffer (100 mM NaCl, 25 mM Tris, 8 mM MgCl_2_, pH 7.5) was added to the pellet. The phage suspension was centrifuged again at 13000g for 10 min to separate cell debris. Solid CsCl was added slowly up to a final concentration of 1.40 g/mL, and swirled gently to dissolve in the phage suspension. The phage-CsCl solution was loaded to ultracentrifuge tubes and run 24 h at 200,000 g using a Beckman 70.1 Ti rotor. The phage band was transferred to a 10 kDa cutoff dialysis cassette and dialyzed three times against 500 mL of gelatin-free SM buffer each. Finally, the phage preparation was filtered (0.45 μm) and stored at 4°C.

### Real-time PCR.

Bacteria were grown at 37°C up to an OD_600_ 0.5 in LB medium. The culture was infected with ɸ29 at an MOI of 10. All infected strains were incubated for 10 min at 24°C to allow phage attachment, then placed in 37°C shaker and timed. 500 μL aliquots of the B. subtilis cultures were withdrawn every 15 min. For analysis of the internal and external phage DNA concentration, cells and supernatants were separated by centrifugation at 3,800 g for 2 min and pellets were resuspended in 500 μL buffer. 50% chloroform (V/V) was added to interrupt the phage infection process and samples were centrifuged at 16,000 g for 10 min to remove cell debris. Analysis of the DNA samples were performed by real-time PCR in a Bio-Rad 96-well real-time thermocycler (iCycler). For viral DNA amplification following oligonucleotides were used: ɸ29-*gp8*-F: GTCAGGGCGATAACTTCA and ɸ29-*gp8*-R: TACGATCAACAAGGGACG. The data obtained for each DNA sample was interpolated to a standard curve constructed with known amounts of purified, full-length ɸ29 DNA. ɸ29 DNA was isolated using Invitrogen PureLink genomic DNA kit.

### ɸ29 lysis test and lysogenic SPβ lysis test.

In a ɸ29 lysis test, B. subtilis strains were freshly grown to an OD_600_ of 0.5 in LB medium, then mixed with phage ɸ29 at an MOI of 1.0 and incubated at 37°C for 1 h. External (released) phages were collected by centrifugation at 3,800 g for 2 min. For the entire assembled phages (including the phages still inside the host cells), the samples were mixed with 1% chloroform (final concentration), then mixed by 10 tube inversions, and finally centrifuged. A spot assay was performed on the lawn of a *dynA*-knockout *168* strain to measure the phage titers. For the SPβ lysis tests, 5 μg/mL mitomycin was added to the bacterial cultures of wild-type and *dynA*-knockout strains. Cultures were shaken at 37°C for 30 min. After incubation, mitomycin was washed away with fresh LB medium. Lysed SPβ were counted using a quantitative spot assay that was performed on the lawn of the SPβ-susceptible *25152* strain carrying the *dynA::tet* allele (3).

### Phage-capsid staining.

CsCl-purified phage (500 μL) were mixed with 5 μL Alexa Fluor 647 (Succinimidyl Ester, 1 mg/mL in DMSO) and rotated for 1 h at 24°C. 1 M NaCl and 10% PEG-8000 were sequentially added to the solution and stirred for at least 30 min. The mixture was then centrifuged for 10 min at 13,000 g. The pellet was resuspended in gelatin-free SM buffer (>1/5 of the original volume) and centrifuged again at 13,000 g for 10 min to obtain the supernatant. Labeled phages were separated from unbound dye using Illustra NAP-5 columns. Columns were equilibrated with 10 mL gelatin-free SM buffer and then load 500 μL of the supernatant. Every drop (∼50 μL) was collected in 200 μL PCR tubes and fluorescence intensity and phage activity was assayed using an Infinite200 PRO (Tecan, Grödig, Austria) fluorescent plate reader. Specially, 2 μL of drops were, respectively, added to 198 μL gelatin-free SM buffer in a 96-black plate and the fluorescence intensity (630/670 nm [Ex/Em]) Was measured. Another 2 μL of each fraction was added to 198 μL of a exponentially growing bacillus culture (OD_600_ = 0.5) in a 96-transparent plate and OD values were measured for 6 h with 30-min intervals. Fractions containing active phages with high fluorescence based on the capsid labeling were collected and stored in dark at 4°C.

### Phage-DNA staining.

CsCl-purified phages (500 μL) was mixed with 5 μL Hochest (1 mg/mL in H_2_O) and rotated for 1 h at room temperature. 1 M NaCl and 10% PEG-8000 were sequentially added to the solution and stirred for at least 30 min. The mixture was then centrifuged at 13,000 g for 10 min. The pellet was resuspended in gelatin-free SM buffer (>1/5 of the original volume) and centrifuged again at 13,000 g for 10 min to obtain the supernatant. The NaCl/PEG cleanup was repeated twice.

### Fluorescent microscopy.

B. subtilis strains were grown up to an OD_600_ of 0.5–1.0 in fresh LB medium at 37°C. Labeled ɸ29 and DynA-GFP were visualized on a Delta Vision Elite (GE Healthcare) equipped with an Insight SSI illumination (Insight Lighting, Rio Rancho, NM, USA) and a CoolSnap HQ2 CCD camera (Teledyne Photometrics, Tucson, AZ, USA). Images were taken with an oil immersion PSF U-Plan S-Apo 1.4 NA objective. A 3-color standard set Insight SSI unit with excitation wavelengths (blue 390/18 nm, green 475/28 nm, far-red 632/22 nm), single bandpass emission wavelengths (blue 435/48 nm, green 573/36 nm, far-red 679/34 nm), and a suitable polychroic for blue/green/far-red were used. ImageJ (42) was used to analyze the micrographs.

### Single-molecule tracking.

B. subtilis 168 DynA-mVenus cells were grown in S7_50_ minimal medium at 30°C under shaking conditions to an OD of 1. Afterwards, the cells were infected with ɸ29 bacteriophages for 1 h, with a multiplicity of infection (MOI) of 1. Cells were spotted on coverslips (25 mm, Marienfeld) and covered with an agarose pad 1% (wt/vol), made of S7_50_ Medium and a smaller coverslip (12 mm, Marienfeld).

Imaging was performed with a Nikon Eclipse Ti microscope equipped with a high numerical aperture objective (CFI Apochromat TIRF 100XC Oil, NA 1.49), an EM-CCD camera (ImagEM X2, Hamamatsu) and a YFP filter set (BrightLine 500/24, Beamsplitter 520 and BrightLine 542/27). mVenus fluorophores were excited by the central part of a laser beam (TOPTICA Beam Smart, 515 nm, max. power 100 mW) with a laser intensity of 20 mW, corresponding to about 160 W/cm^2^. Each movie was recorded with an integration time of 20 ms via stream acquisition, using Nikon NIS-Elements BR. Movies consist of 3500 frames. Because single molecule level was reached after about 500 frames, the first 500 were discarded before analysis.

### Single-molecule tracking—data analysis.

First, the videos were cut with Fiji (ImageJ) (42) and the last 1000 frames were used. Afterwards, the cell meshes were set with oufti (43). For particle detection, U-track (44), a MATLAB software, was used. Here, the minimal length of tracks was set to 5 and to link to points, no gaps for the particle detection was allowed. With the MATLAB software SMTracker (33) the data were analyzed. Thereof the import panel -for localization and *dwell times* - and the Gaussian mixture model (GMM) analysis panel were used.

### Statistical analysis.

We used the R suite program to calculate statistical significances (45).
