# Supplementary figures and images for: A Bacterial Dynamin-Like Protein Confers a Novel Phage Resistance Strategy on the Population Level in Bacillus subtilis
Source: mBio. 2022 Feb 15;13(1):e03753-21. doi: 10.1128/mbio.03753-21 (PMC8844932; doi:10.1128/mbio.03753-21)

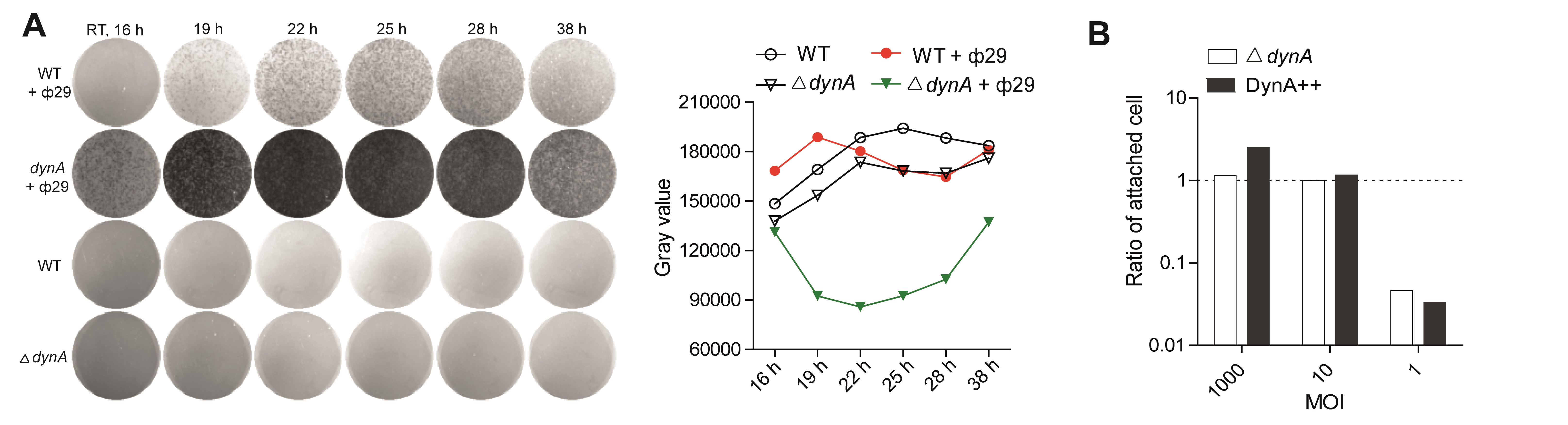

Supplement: FIG S1 [file mbio.03753-21-sf001.tif]

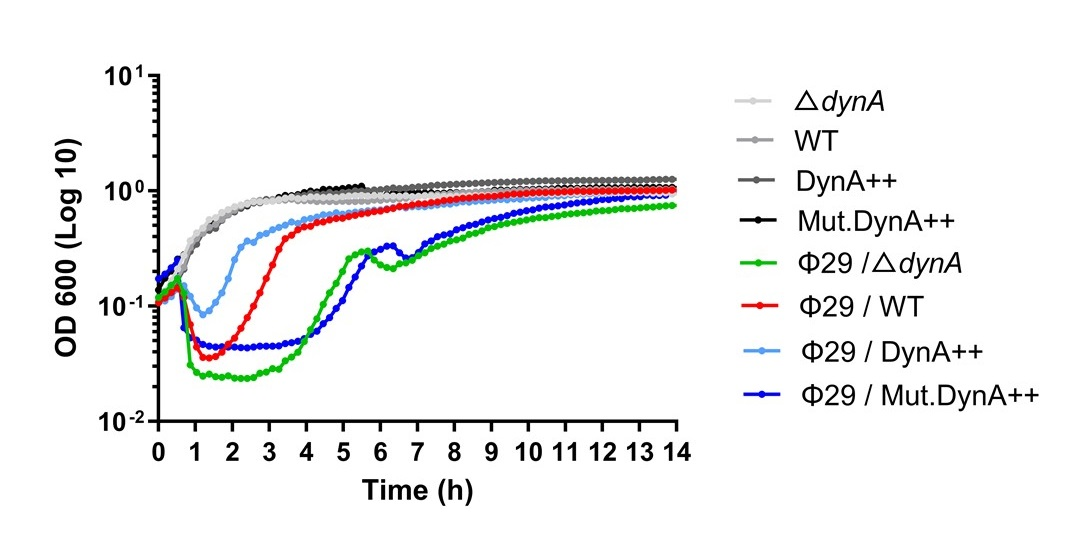

Supplement: FIG S2 [file mbio.03753-21-sf002.tif]

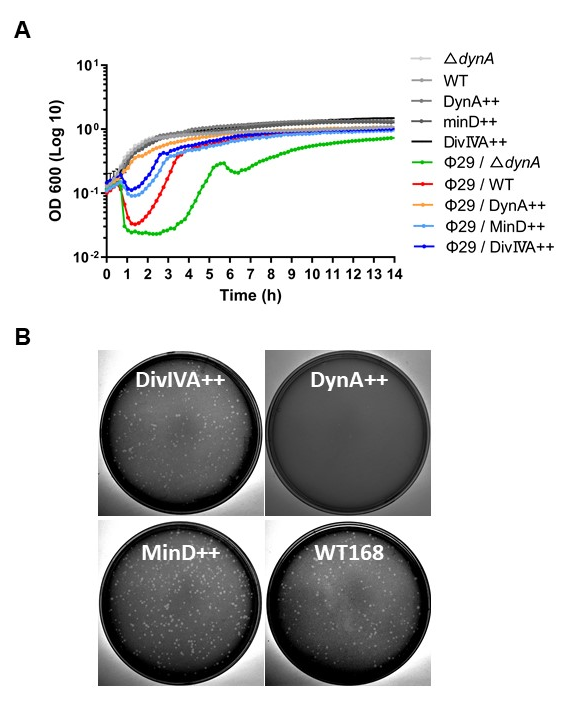

Supplement: FIG S3 [file mbio.03753-21-sf003.tif]

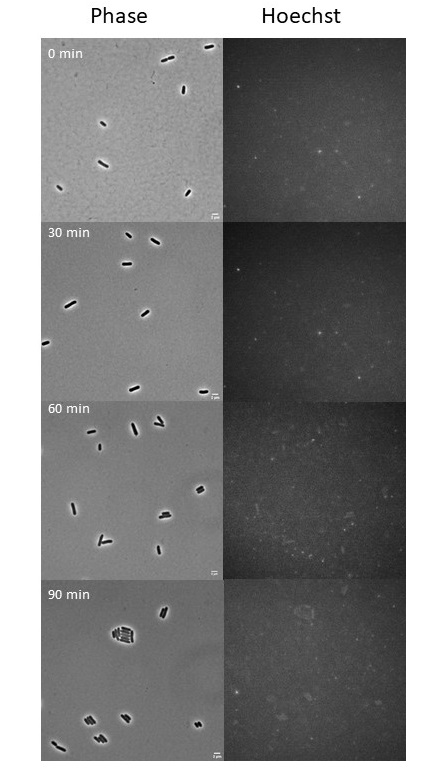

Supplement: FIG S4 [file mbio.03753-21-sf004.tif]
